# Hospital Meetings

**Published:** 1904-12-03

**Authors:** 


### HOSPITAL MEETINGS.

### THE MIDDLESEX HOSPITAL.

At the Quarterly Court held on Thursday, November 24th, under the chairmanship of H.R.H. Prince Francis of Teck, it was stated that Lord Sandhurst, owing to increased calls upon his time, had resigned the chairmanship of the weekly board, a position he had held intermittently for many years. He had been succeeded by Lord Cheylesmore, a member for many years. Prince Francis of Teck had been appointed a vice-president. One of the wards of the hospital had been closed for four months, while the walls were lined with crystopal, at the expense of Mr. L. Bischoffsheim; and other improvements, including the replacing of certain old boilers by Berryman heaters, had been effected. The Dresden legacy of £27,300, for the assistance of patients leaving the hospital; £2,501 5s. from the Metropolitan Hospital Sunday Fund for the General and Convalescent Home Funds; and £864 10s. 4d. from the David Hughes Trust Fund had been received. The increased work in the Out-patient Casualty Department had rendered the appointment of a clerk for that department necessary. The number of patients treated in the hospital during the six months ending September 30th was 2,064, and there had been 23,678 out-patients. The Ladies' Committee in connection with the recent dinner had collected for the Cancer Fund a sum of £2,000; and the Metropolitan Hospital Sunday Fund had made a grant of £268 6s. 8d. for the same purpose. Over 1,000 patients had been admitted to the convalescent home.

It had been found necessary to provide accommodation for the teaching of physics, and the old pathological laboratory had been reconstructed for the purpose at a cost of £180. Mr. A. M. Kellas had been appointed lecturer on physics.

#### Cancer Research.

Dr. Lazarus-Barlow had been reappointed director of the cancer research laboratories, and Messrs. Handley and Douglas, the two research scholars, had been reappointed. Steady progress was being made in elaborating the rich store of information on cancer contained in the hospital records. At the same time other lines of research in cancer were being carried out by the director and the six gentlemen working in the laboratories. An increase of laboratory accommodation for this important work was urgently needed. The investigators themselves were inconveniently crowded, and offers of voluntary assistance had had to be regretfully declined by the director owing to lack of space The third report from the laboratories was published in July. It contained a full record for the year 1903. Fair progress had already been made in compiling the report for 1904, and it was hoped that this would be issued during the spring of 1905.

#### Clinical and Bacteriological Laboratories.

A considerable amount of work for the hospital was now carried out in the clinical and bacteriological laboratories, 1,050 specimens from the wards and operating theatre having been examined and reported on between January 1st and October 31st of the present year, the investigations being in many cases of an elaborate nature and occupying much time.

The following investigations had been brought to completion during the last three months and the results would be published shortly:—(1) A research into the method of the action of the "high frequency" electric currents which are used in the treatment of various forms of disease, and (2) an investigation into the causes of puerperal fever—an extensive research which had occupied nearly three years.

Papers dealing with other aspects of research work carried on in the clinical and bacteriological laboratories would form the bulk of the forthcoming Volume IV. of "The Middlesex Hospital Archives."

#### Electrical and Light Department.

The total number of cases was 9,885. There were 568 radiant heat baths given to rheumatic and skin-disease cases; 113 *x* ray screen examinations were made for accident and out-patient cases; 308 skiagrams were taken. The cancer, medical, surgical, and skin-disease cases had all increased in number, and at times there was difficulty in finding space to deal with them.

The report was adopted unanimously, and a vote of thanks to the chairman closed the proceedings.

### ROYAL HOSPITAL FOR INCURABLES, PUTNEY HEATH.

The jubilee annual meeting and the one hundred and first half-yearly election of candidates of the Royal Hospital for Incurables, Putney Heath, took place on Friday in last week in the Grand Hall of the Cannon Street Hotel, E.C. The number of people present was very small indeed. It included, however, the following members of the Board of Management: Mr. H. J. Allcroft (Treasurer), Sir William Clarke, Mr. Thomas Conway, Mr. James Dix, Mr. Henry Ellis, Mr. T. Clarence E. Goff, Colonel Holland, Mr. J. M. Rücker, Mr. David H. Small, Captain G. A. Webbe, Mr. Thomas Wickham, and Mr. J. Turton Wright.

Mr. H. J. Allcroft presided, and in proposing the adoption of the annual report—which was taken as read—he referred to the financial position of the hospital. He regretted to announce a large reduction in income, particularly in legacies which alone show a falling off of nearly £9,000. In consequence only thirty candidates could be elected as against thirty-five at the last election.

The adoption of the report having been carried unanimously, Captain Webbe proposed that the following members of the Board of Management, retiring by rotation, offer themselves for re-election, viz.: Sir William Clarke, Admiral Campbell, Sir F. Dixon-Hartland, M.P., Colonel Holland, Mr. David H. Small, Mr. Thomas W. Wickham, and that the two following members, having been added by the Board to fill vacancies, offer themselves for election: Mr. J. Turton Wright, Mr. G. M. Rutherford.

This resolution, seconded by Mr. Ellis, was carried.

Mr. Daniel Stock, speaking from the body of the hall, remarked upon the very small audience. The chairman, in reply, said that by the very few people present it might be concluded that things were going on smoothly in the hospital.

The meeting then terminated and the election of candidates commenced. Among the one hundred and twenty-seven applicants for the thirty vacancies were a "hospital nurse," a "trained nurse," and a "Mildmay deaconess."

